# *Wolbachia* strain *w*Pip yields a pattern of cytoplasmic incompatibility enhancing a *Wolbachia-*based suppression strategy against the disease vector *Aedes albopictus*

**DOI:** 10.1186/1756-3305-5-254

**Published:** 2012-11-12

**Authors:** Maurizio Calvitti, Riccardo Moretti, Amanda R Skidmore, Stephen L Dobson

**Affiliations:** 1 Laboratory of Sustainable Management of the Agro-ecosystem, ENEA (Italian National Agency for New Technologies, Energy and Sustainable Economic Development), Via Anguillarese, 310, 00123, Rome, Italy; 2 Department of Entomology, University of Kentucky, Lexington, KY, 40546, USA

**Keywords:** Cytoplasmic incompatibility, *Wolbachia pipientis*, Incompatible insect technique, Transinfection, *Aedes albopictus*

## Abstract

**Background:**

Cytoplasmic incompatibility (CI) is induced in nature by *Wolbachia* bacteria, resulting in conditional male sterility. Previous research demonstrated that the two *Wolbachia* strains (*w*AlbA and *w*AlbB) that naturally co-infect the disease vector mosquito *Aedes albopictus* (Asian tiger mosquito) can be replaced with the *w*Pip *Wolbachia* strain from *Culex pipiens.* Since *Wolbachia*-based vector control strategies depend upon the strength and consistency of CI, a greater understanding is needed on the CI relationships between *w*Pip, *w*AlbA and *w*AlbB *Wolbachia* in *Ae. albopictus*.

**Methods:**

This work consisted of a collaborative series of crosses carried out in Italy and in US to study the CI relationships between the “*w*Pip” infected *Ae. albopictus* strain (AR*w*P) and the superinfected SR strain. The *Ae. albopictus* strains used in Italian tests are the *w*Pip infected AR*w*P strain (AR*w*P_IT_), the superinfected SR strain and the aposymbiotic AR strain. To understand the observed pattern of CI, crossing experiments carried out in USA focused on the study of the CI relationships between AR*w*P (AR*w*P_US_) and artificially-generated single infected lines, in specific HTA and HTB, harbouring only *w*AlbA and *w*AlbB *Wolbachia* respectively.

**Results:**

The paper reports an unusual pattern of CI observed in crossing experiments between AR*w*P and SR lines. Specifically, AR*w*P males are able to induce full sterility in wild type females throughout most of their lifetime, while crosses between SR males and AR*w*P females become partially fertile with male aging. We demonstrated that the observed decrease in CI penetrance with SR male age, is related to the previously described decrease in *Wolbachia* density, in particular of the *w*AlbA strain, occurring in aged superinfected males.

**Conclusions:**

The results here reported support the use of the AR*w*P *Ae. albopictus* line as source of “ready-made sterile males”, as an alternative to gamma radiation sterilized males, for autocidal suppression strategies against the Asian tiger mosquito. In addition, the age dependent CI weakening observed in the crosses between SR males and AR*w*P females simplifies the downstream efforts to preserve the genetic variability within the laboratory AR*w*P colonies, to date based on the antibiotic treatment of wild captured superinfected mosquitoes, also reducing the costs.

## Background

*Aedes* (*Stegomyia*) *albopictus* (Diptera: Culicidae) (Asian tiger mosquito) is known as a mosquito species with an invasive behavior and a competent vector of various dangerous viruses [1-3]. In a few years since its arrival in Italy, *Ae. albopictus* has gained the position of the most important public health vector species and is at the top of the noxious species list [4,5]. The recent occurrence of autochthonous epidemics of Chikungunya and Dengue viruses in southern Europe [6,7] transmitted by *Ae. albopictus,* seems to confirm that the currently applied mosquito control methods (larval control, source reduction, and community participation) are not sufficient to keep the mosquito adult density below the epidemic risk threshold [8]. This was the main reason that stimulated the start of research for the development of a Sterile Insect Technique (SIT) program in Italy [9]. Several characteristics make *Ae. albopictus* a suitable candidate for SIT application, as follows: it is a recently introduced species with population showing a low genetic variability [10], it mainly colonizes urban areas while showing low aptitude to establish in rural and natural areas, it has a low active dispersal activity [11-13], and it is relatively easy to manage under mass rearing and artificial conditions [9]. Nowadays interest in SIT for vector control has resurfaced, driven also by the availability of new technologies that have the potential to provide significant improvements in cost-effectiveness for SIT [14]. Although males of *Ae. albopictus* may be sterilised through ionizing radiations, by exposing mature pupae to a dose of 30–40 Gy γ-rays [15], the technique requires laborious handling procedures to prepare pupae for irradiation and transportation, in addition to the need for a radiation source, which is an expensive tool that needs an infrastructure requiring a substantial regulatory framework. Developing alternative technologies to produce “ready-made sterile males”, avoiding sterilization with gamma rays, could improve the overall competitiveness of the released insects with a consequent improvement in program efficiency and a significant decrease in costs.

In the last two decades, scientists have given an increasing level of attention to *Wolbachia pipientis* Hertig (Alphaproteobacteria, Rickettsiales) [16], a widespread intracellular bacterium [17] able to manipulate host reproduction [18]. Cytoplasmic incompatibility (CI) is the most commonly detected type of *Wolbachia*-induced reproductive alteration in insects [19]. When a population contains individuals with different *Wolbachia* infection types (infected/uninfected or infected by different *Wolbachia* strains) their crosses can be (i) compatible and produce viable offspring; (ii) incompatible in both directions and produce infertile eggs (a phenomenon called bidirectional CI) or (iii) incompatible in one direction while the reciprocal cross is fertile (unidirectional CI). While the genetic and biochemical mechanisms of CI are not known, the cytological effects are clear [20]. Sperm that are “modified” by *Wolbachia* in the testes show abnormal processing following fertilization of the egg, if the appropriate *Wolbachia* strain is not present in the egg to “rescue” the modification [21].

These attributes are now being studied by many research groups with the aim of developing new technologies and strategies to achieve significant improvements in pest and vector control. *Wolbachia*-mediated CI has been proposed as a strategy for insect control via two approaches: (1) using CI to cause sterility for a mass male release strategy analogous to sterile insect technique and consequently named “Incompatible Insect Technique” (IIT) [14,22-25], or (2) using the reproductive advantage afforded by *Wolbachia*-induced CI as a tool for a population replacement strategy, driving desired phenotypes (*e.g*., lower affinity for pathogens) into medically important mosquito populations [26-29]. Both of these approaches require a method to artificially transfer *Wolbachia,* generating new patterns of CI [30]. In 2010, a transinfected line (AR*w*P) of *Ae. albopictus* was generated by removing the naturally occurring co-infection of *w*AlbA plus *w*AlbB and microinjecting in the aposymbiotic eggs the *w*Pip *Wolbachia* strain from *Culex pipiens molestus* (Diptera: Culicidae) [31]. The new symbiosis was shown to be stable and efficiently transmitted from females to their offspring. Since *Wolbachia*-based vector control strategies rely on the strength and consistency of CI, a greater understanding is needed of the CI pattern resulting between the AR*w*P line and the naturally occurring infection types in *Ae. albopictus*. The main goal of this work was to characterize and explain the pattern of CI displayed in crosses between the AR*w*P and SR mosquito lines and evaluate its implications in the development of *Wolbachia*-based strategy against the Asian tiger mosquito.

This was done by analyzing the egg hatching in crossing experiments involving five laboratory mosquito lines: i) the transinfected AR*w*P (harbouring *w*Pip); ii) the naturally superinfected SR strain (harbouring both the *w*AlbA and *w*AlbB infection types); iii) the artificially single-infected HTA strain (infected with *w*AlbA only); iv) the artificially single-infected HTB strain (infected with *w*AlbB only); and v) the AR strain that has had its *Wolbachia* infection removed (*i.e.* aposymbiotic). In addition to examining young males, for some strains we evaluated also the effects of male aging on CI, through crosses involving old males.

## Methods

### Mosquito strains and rearing conditions

This work consisted of a collaborative series of crosses: one series (Series I) conducted at the Laboratory of Sustainable Management of the Agro-ecosystems of ENEA (Rome, Italy); the other series (Series II) at the Department of Entomology, University of Kentucky (Lexington, Kentucky, USA). The *Ae. albopictus* strains used in Italian tests are the *w*Pip infected AR*w*P strain (AR*w*P_IT_), the superinfected SR strain and the aposymbiotic AR strain, as defined in a previous work [31]. In order to avoid genetic depression, the three lines have been periodically outcrossed with wild superinfected males, following antibiotic treatment for *Wolbachia* removal [32,33]. The crossing experiments carried out in USA focused on the study of the CI relationships between AR*w*P (AR*w*P_US_) and artificially-generated single infected lines, in specific HTA and HTB, harbouring only *w*AlbA and *w*AlbB *Wolbachia* respectively. AR*w*P_US_ colony originated from a stock of about 5,000 eggs produced by the AR*w*P_IT_ strain and shipped to the University of Kentucky in 2010.

All colonies, both in Italy and USA, were maintained as previously described [32]. Since temperature of water used for larval rearing may influence *Wolbachia* density and CI penetrance [34], care was used to keep the water temperature between 25 and 27°C.

When testing for CI, potential confounding effects influencing fertility of the crossing experiments, such as the nuclear background of the host, have to be limited by the experimental design [35]. Since AR*w*P_US_ and HTA-HTB had been generated from wild type mosquito strains having a different geographic origin, HTA and HTB lines (originated from US “Hou” strain) and AR*w*P_US_ were outcrossed for 5 consecutive generations with aposymbiotic AR males obtained from a stock of eggs also shipped to US from Italy.

The HTA line was generated using a previously described microinjection procedure [36]. In brief, aposymbiotic embryos (HT1 strain) [32] were microinjected with cytoplasm containing *Wolbachia* from wild type *Ae. albopictus* embryos (Hou strain, Texas 1986). Adult females developing from microinjected eggs were mated with aposymbiotic males (HT1 strain), blood fed, isolated and allowed to oviposit individually. Iso-female lines were generated from hatching egg broods that originated from females in which *Wolbachia* was detected. This procedure was repeated until maternal transmission rates of *Wolbachia* reached 100% for more than three generations. For the HTA strain, selection was repeated for eight generations. After the second generation, all iso-females tested were PCR positive for *Wolbachia* using the *Wolbachia* molecular diagnosis protocol described below. Additional tests with clade-specific primers demonstrated that infected females were positive for A-clade specific *Wolbachia* (328 F, 691R primer set). All tests using B-clade specific primers (183 F, 691R primer set) were negative, indicating the loss of one of the *Wolbachia* types. Following the eighth generation, iso-female selection was stopped and the line was maintained using a generation specific (non-overlapping) rearing scheme in which no selection was used. Periodic (every three generations) A-clade specific *Wolbachia* primers checks were performed to ensure the stability of *Wolbachia* infection levels. HTB line was obtained following a similar procedure, as described in a previous work [37].

### *Wolbachia* molecular diagnosis

PCR assays were performed to check that all males and females mosquitoes used in the experiments had the expected infection type. Molecular discrimination of uninfected from infected males was performed by the diagnostic *wsp* primers (81 F-691R) that amplify a region of the gene encoding the *Wolbachia* outer surface protein (*wsp*) and allow for a broad identification of *Wolbachia* strains [38]. *Wolbachia* strains *w*Pip and *w*AlbB can be easily identified by the same specific set of primers (183 F, 691R) [39] when they live separated in their natural hosts (*Cx. pipiens* and *Ae. albopictus* respectively). Here we were faced with the need to discriminate *w*Pip from *w*AlbB infected individuals to ascertain the absence of contaminations. For this purpose we designed the following specific set of primers: *w*PF (5^′^- CGACGTTAGTGGTGCAACATTTA -3^′^) and *w*PR (5^′^ AATAACGAGCACCAGCAAAGAGT-3^′^) by which we were able to specifically amplify the *wsp* region of the *w*Pip *Wolbachia* strain.

DNA was extracted from individual mosquitoes by dissecting and homogenizing ovaries or testis of adults in 100 μl STE with 0.4 mg/ml proteinase K [4]. The PCR cycling procedure used was: 94°C for 5 min followed by 35 cycles of 94°C for 30 s, 55°C (54°C for the *w*P primers) for 30 s, 72°C for 40 s and a single final step at 72°C for 10 min. Amplified fragments were electrophoresed on 2% agarose gels, stained with ethidium bromide (1 μg/ml) and visualized under ultraviolet light. DNA template quality was assessed by amplifying a fragment of the insect mitochondrial cytochrome oxidase I (COI) DNA, using the primers CI-J-1751 and CI-N- 2191 [40].

### Crossing experiments

The following series of 7 crosses (female x male) were set up in Italy: 1) SR x SR, 2) AR*w*P_IT_ x AR*w*P_IT_, 3) AR x AR, 4) AR x SR, 5) AR*w*P_IT_ x SR, 6) AR x AR*w*P_IT_, 7) SR x AR*w*P_IT_.

CI relationships between AR*w*P, SR and AR mosquito strains had been previously observed and shown to result in a pattern of bidirectional incompatibility for crosses between AR*w*P and SR mosquito lines and unidirectional CI by crossing AR*w*P with AR and SR with AR [31-33]. In this work, we examined the variable "male age" for crosses of SR and AR*w*P males with virgin 2–4 d old SR, AR*w*P and AR females. For the objectives of this work, female age was kept constant in all crosses (2–4 d).

For Series I experiments male age was assessed from the emergence (= Day 0). Groups of males were aged 3, 11, 19 and 27 days (± 1). For each cross type, 20 females and 20 males were kept together in mating cages (40 × 40 × 40 cm) over a 24 h period. Subsequently, female groups were allowed to feed on anesthetized mice, in accordance with the Bioethics Committee for Animal Experimentation in Biomedical Research and following procedures approved by the ENEA Bioethical Committee. Gravid females were then removed from the mating cages and transferred to a new cage (oviposition cage) and provided with oviposition devices as previously described [31]. Eggs were counted and stored for 5 d before allowing them to hatch, by the immersion in a nutrient broth stimulating hatching [9]. In crosses with no egg hatch, females were dissected to check for the presence of spermatozoa. Males were kept in the original cages. After removing the first groups of females, a new cohort of virgin females was added to the cages containing males at 1:1 female:male ratio. Following this procedure, the CI relationship between the different *Wolbachia* infections was investigated under conditions similar to those common to a release of young incompatible males, getting older, more experienced and consuming their sperms in the field. This cycle was repeated for the four male age-classes.

The general protocol described above was used also for Series II experiments set up to study the CI relationships between the AR*w*P line and the two single-infected strains HTA and HTB. The following 7 crosses (female x male) were set up in US: 1) AR*w*P_US_ x HTA, 2) HTA x AR*w*P_US_, 3) AR*w*P_US_ x HTB, 4) HTB x AR*w*P_US_, 5) AR*w*P_US_ x AR*w*P_US_, 6) HTA x HTA, 7) HTB x HTB. The design of the latter crosses did not include male age and all crosses consisted of young (2–4 d old) males. Four cage replications were set up for each crossing type.

At the end of the crossing experiments, the infection status of the males was checked (by PCR assays) to verify that all had the expected infection type (to avoid an incorrect interpretation of the results possibly due to the rare presence of aposymbiotic individuals among males of an infected line). Molecular analysis was also performed on any males found dead in the cages to determine their symbiotic status.

### CI computation and statistics

Calculation of CI expression was based upon the mean egg hatch rate found in incompatible crosses in comparison with the results from the compatible crosses (e.g. AR*w*P x AR*w*P, SR x SR, HTB x HTB, HTA x HTA), using the CI_corr_ index [41]. This index allows the exclusion of embryonic mortality observed in compatible crosses and male age effects that are not due to CI expression.

Within the different crossing types egg-hatching data and CI values were compared in relation to male ages. Normality of egg-hatching data was examined by D’Agostino and Pearson omnibus normality test using Prism 5 (Graphpad software). Significant differences among mean egg hatch rates were tests by analysis of variance (ANOVA) on arcsin sqrt transformed data. A statistical comparison was then performed by Newman-Keuls Multiple Comparison Test (α = 0.05). Paired *t* test was also used to analyze differences in mean egg hatching rates between two different groups.

## Results

### Male age effects on hatch rate and CI expression

In all crosses between males and females harbouring the same *Wolbachia* infection type, the mean hatch rate was initially high (80.0 ± 6.0% to 86.2 ± 4.7% hatch) (Table 1). However, starting from the 3rd male age class, we observed a significant decline in egg hatch within each of the compatible crosses. In the 19 ± 1 d male age class, egg hatch had significantly fallen to 46.4 ± 10.3 0% in the AR*w*P_IT_ (ANOVA: *F* = 16.65; d.f. = 12; *P* < 0.001), to 47.7 ± 7.5% in AR (ANOVA: *F* = 10.69; d.f. = 12; *P* = 0.001) and to 63.2 ± 10.2% in SR mosquito line (ANOVA: *F* = 23.01; d.f. = 12; *P* < 0.001). A further decrease was observed with the males of the fourth age class. This general trend agrees with a gradual decrease of the insemination capacity as males get older already reported in previous works [33-42].

**Table 1:** **Percent egg hatch from crosses between *Ae. albopictus *lines, in dependence of male aging**

**Cross type (♀ X ♂)**	**Percent egg hatch (mean ± SD) at different male ages (days ± 1)**			
	**3 d**	**11 d**	**19 d**	**27 d**
SR x SR	86.2 ± 4.7 (2758)	79.1 ± 7.9 (3001)	63.2 ± 10.0^a^ (2476)	45.8 ± 9.9^b^ (2238)
AR*w*P_IT_ x AR*w*P_IT_	84.3 ± 7.9 (2646)	69.9 ± 8.0 (2960)	46.4 ± 10.0^a^ (2253)	38.0 ± 15.8^a^ (1944)
AR x AR	80.0 ± 6.0 (2344)	70.0 ± 12.3 (1999)	47.7 ± 7.5^a^ (2843)	43.0 ± 15.4^a^ (1822)
AR x SR	0.0 (2730)	0.0 (2738)	0.5 ± 0.7 (3034)	1.0 ± 2.0 (2258)
AR*w*P_IT_ x SR	0.0 (3142)	4.0 ± 0.8^a^(2754)	18.0 ±2.4^b^ (2456)	19.2 ± 8.1^b^ (2678)
AR x AR*w*P_IT_	0.0 (2669)	0.3 ± 0.5 (3453)	0.2 ± 0.2 (2113)	0.0 (2014)
SR x AR*w*P_IT_	0.0 (3681)	0.0 (2905)	1.3 ± 1.9 (2882)	0.0 (2251)

Presented data show the results of the Series I experiments, carried out in Italy.

SR= *w*AlbA + *w*AlbB superinfected; AR*w*P_IT_ =*w*Pip infected; AR = aposymbiotic.

Number of total scored eggs are in parenthesis. Within a row, letters following the data indicate significant differences (P < 0.05) (Anova-Newman-Keuls Multiple Comparison Test).

**Figure 1:**
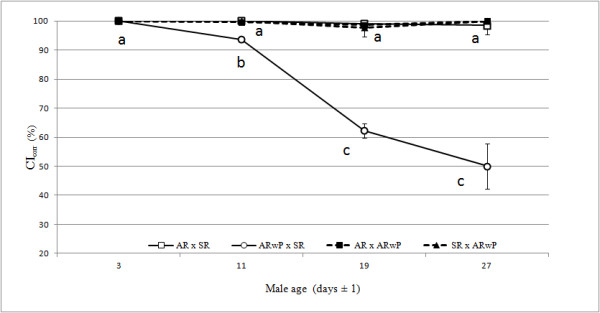
**Estimated CI levels in function of male ageing, CI**_**corr **_**(%) **[41]** was estimated from egg hatching data observed in incompatible and compatible crossing types (female x male) involving SR (superinfected), AR (aposymbiotic) and AR*w*P**_**IT **_**(transinfected) *Ae. albopictus *lines.** Different letters at each male age interval indicate significant differences (*P*<0.05) (Anova-Newman-Keuls Multiple Comparison Test).

As shown in Table 1, all the crosses involving males of the first age class and females with a different infection status were characterized by a complete egg hatch failure. This result did not significantly change with male aging in three out of four incompatible crosses. Only in the cross between AR*w*P_IT_ females and SR males the percentage of eggs hatching increased significantly starting from the second age class (ANOVA: *F* = 150.00; d.f. = 12; *P* < 0.001) and reaching values close to 20% with the oldest males. Consequently in this cross type CI_corr_ decreased to about 50% of that observed with 3 days old males (100%) (Figure 1).

### CI in crosses between single-infected lines

The mean egg hatch values observed in the AR*w*P_US_ compatible crosses of Series II were lower than that observed in the Series I crosses (45.0 ± 22.2 and 84.3 ± 7.9% respectively at the first age class) (Table 2). A low hatch rate (44.7 ± 13.7%) was also observed in compatible crosses of the HTA line while HTB was the single infected line showing the highest egg fertility (66.0 ± 12.4%).

**Table 2:** **Percent egg hatch and CI**_**corr **_**level from crosses between *Ae. albopictus *lines different by their *Wolbachia *infection status**

**Cross type (♀ X ♂)**	**Percent egg hatch**	**CI**_**corr **_**level**
	**(mean ± SD)**	**(mean ± SDCP**
AR*w*P_US_ x AR*w*P_US_	45.0^a^ ± 22.2 (3210)	0
HTA x HTA	44.7^a^ ± 13.7 (2976)	0
HTB x HTB	66.0^b^ ± 12.4 (2165)	0
AR*w*P_US_ x HTA	0.9^c^ ± 0.6 (3266)	98.0^a^ ± 1.5
HTA x AR*w*P_US_	0.0^c^ (3165)	100^a^
AR*w*P_US_ x HTB	19.9^d^ ± 5.5 (2076)	55.8 ± 8.4^b^
HTB x AR*w*P_US_	0.0^c^ (3241)	100^a^

Presented data show the results of the Series II experiments, carried out in USA, using 3 ± 1 days old males. AR*w*P_US_ = *w*Pip infected; HTA = *w*AlbA single infected, HTB = *w*AlbB single infected. Number of total scored eggs are in parenthesis.

Within a column, letters following the data indicate significant differences (P < 0.05) (Anova-Newman-Keuls Multiple Comparison Test).

As shown in Table 2, crosses between the AR*w*P_US_ strain and the single infected lines showed low egg hatch levels and high CI_corr_ values (close to 100%) with one exception, in the cross between AR*w*P_US_ females and males with the *w*AlbB infection only (*i.e.* HTB line) CI_corr_ weakened to 55.8% (Student’s test, *α* = 0.05; *t* = 2.03), corresponding to a 19.9 ± 5.5 percentage of eggs hatching. Reciprocal crosses between individuals with the *w*Pip (AR*w*P_US_) and *w*AlbA (HTA) infections were bidirectionally incompatible. In contrast, reciprocal crosses between individuals with the *w*Pip (AR*w*P_US_) and *w*AlbB (HTB) infections showed full incompatibility in one direction only.

## Discussion

Data obtained by studying the egg hatch rate and thus computing the level of CI in crosses between AR*w*P_IT_ and SR lines have provided interesting insights that suggest an unusual pattern of bidirectional CI, changing partially to unidirectional as superinfected SR males get older. We observed that males harbouring the *w*Pip *Wolbachia* strain remain strong CI inducers, despite their age and regardless of whether they mate with naturally superinfected or uninfected females. In contrast, in crosses of naturally-superinfected males (SR line) with AR*w*P_IT_ females, CI drops so that egg hatching increases to approximately 20% as males reach the fourth age class (Table 1).

A number of studies have documented that the strength of *Wolbachia*-mediated CI can decrease as males get older. For example, in *Drosophila melanogaster* Meigen (Diptera: Drosophilidae) this occurs with values ranging from 70-100% of CI expression, when the males are very young (1–2 days), to extremely low levels (4-5% of CI) after males age 15 days [43]. The underlying mechanistic hypothesis is that *Wolbachia* density decreases with male aging [44,45]. In *Cx. pipiens,* CI strength was not found to decrease with male age [46], while an increasing bacterial density was observed in the testes of older males [47], in disagreement with the model according to which the CI penetrance tends to decrease in old males directly proportional to the density of *Wolbachia* in the testes or sperm cysts in general [48].

With regard to *Ae. albopictus,* the data obtained in the present work confirm that the naturally superinfected males express a strong level of unidirectional CI towards the aposymbiotic AR line which does not undergo decreases with male aging, at least up to 26–28 days. In contrast, the same males show age dependent weakening of induced CI when crossed with AR*w*P_IT_ females (Figure 1). Data on CI relationships between SR and AR lines are consistent with previous reports [49] in which a strong CI expression was observed in all crosses between wild superinfected males and laboratory-reared uninfected or *w*AlbA (KOH line) infected young females. In addition, the same authors also reported a pronounced weakening of CI in old KOH males mated with aposymbiotic females. The KOH line is a natural single-infected line harbouring the *w*AlbA *Wolbachia* strain, similar to the artificial HTA described here. While the *w*AlbB density in *Ae. albopictus* remains constant over the mosquito life-time, the density of the *w*AlbA infection decreases gradually as the males get older [50,51].

In this work, we associated the decrease of CI observed in crosses between AR*w*P_IT_ females and SR aged males to the above described *w*AlbA strain-dependent changes in *Wolbachia* density. However, to support this hypothesis, we needed to ascertain that males single-infected by the *w*AlbB *Wolbachia* were only partially incompatible when crossed with *w*Pip infected females. The change in *Wolbachia* density may explain the observed CI pattern if (A)B ≈ B and if the *w*AlbB infection alone causes partial incompatibility.

As an initial test to validate this model, we conducted crosses of single infected *Ae. albopictus*, artificially generated through microinjection, such that individuals carried either the *w*AlbA or *w*AlbB infection, with the AR*w*P_US_ line (Series II crosses). Consistently with model prediction, the *w*AlbA infected males were observed to cause strong CI in crosses with *w*Pip infected females, while the *w*AlbB infection caused partial CI in crosses with *w*Pip infected females. In the reciprocal crosses, males infected with *w*Pip induced strong incompatibility when mated with females that were single infected with either the *w*AlbA or *w*AlbB *Wolbachia* strains. According to the hypothesized model, mean CI_corr_ values found when crossing AR*w*P_IT_ females x old SR males (Figure 1) and ARwP_US_ females x young HTB males (Table 2) were quite similar (49.5± 14.2% vs 55.8 ± 12.8%).

The differences between Series I and II in egg hatch of the AR*w*P line compatible crosses may be explained by a bottleneck resulting from the shipment between laboratories, by the different size of the colonies and by small variations in environmental factors, application of the rearing procedures and handlers. This discrepancy may also reflect an improvement in the ENEA strain quality (mainly occurred in the last two years) resulting from selection, outcrossing practices and enhancement of the rearing methods that will be the topic of a further article. In fact, egg hatching rates of AR*w*P_US_ compatible crosses resemble that reported in a previous work [31] for the AR*w*P_IT_ strain. However, these differences, even if significant, can not invalidate the scientific findings of the whole experimental plan, since the measurement of the CI_corr_ allowed us to take into account the misleading effects of the background mortality while interpreting the results coming from the two Series of experiments. Thus, we thought it was not necessary to wait for the establishment in the USA of AR*w*P colonies showing levels of fertility similar to the Italian colony, since this process could take more than 1 year. Based on our preliminary results, the findings of this work will allow us to set up new high fitness AR*w*P colonies, within 2–3 generations of establishment (Moretti and Calvitti, unpublished data). A lower hatch rate was observed in compatible crosses of the HTA line if compared to egg hatching data reported for other single-infected (*w*AlbA) strains established in other laboratories (i.e. KOH in the Islands of Koh Samui and Mauritius) [32-50]. Since HTA has been recently established in the laboratory, hypotheses to explain this observation include inbreeding effects associated with the establishment of isofemale lines (i.e. increased homozygosis of deleterious loci) and high mortality associated with the artificially generated single *w*AlbB infection type. Introgression with males of uninfected mosquito lines could attenuate potential inbreeding effects as successfully demonstrated with HTB [34].

**Figure 2:**
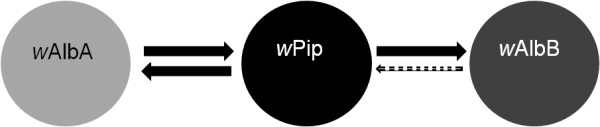
**CI relationships among *w*Pip and *Ae. albopictus *native *Wolbachia *strains.** The *Wolbachia* strain *w*Pip is a strong CI inducer towards *w*AlbA and *w*AlbB (both unable to rescue its mod function) While *w*Pip it is not able to rescue the *w*AlbA mod function, it can partially rescue *w*AlbB (arrows indicate CI intensity).

According to the mod-resc model [20], *w*Pip is able to partially rescue the *w*AlbB mod function, while it is not able to rescue the *w*AlbA mod. Differently, *w*AlbB is a weak CI inducer towards *w*Pip and cannot rescue the *w*Pip mod function (asymmetrical CI). Such asymmetrical CI relationships had been previously reported in the *Cx. pipiens–Wolbachia* system [52,53] as well as for *w*Mel and *w*Ri *Wolbachia* strains [41]. The *Wolbachia* strain *w*AlbA is a strong CI inducer towards *w*Pip and cannot rescue the *w*Pip mod function (Figure 2).

## Conclusions

The reported CI features are consistent with the traits desired for promising use of the AR*w*P line as a suppression tool against *Ae. albopictus*. First, a persistent full CI in older males enforces the efficacy of any CI-based mosquito control strategy. This becomes more relevant given the report that *Ae. albopictus* males in La Réunion show an unexpectedly high mean life expectancy, ranging from 16.2 to 24.5 days [54]. Currently, the optimal radiation doses for a SIT programs against *Ae. albopictus* is chosen in such a way (30 rather than 40 Gy) that it balances induced sterility with the preservation of male competitiveness [15-55]. However, the more the irradiation dose is lowered, the more it is reasonable to assume that a potential recover of fertility could occur when males are still potentially competitive [56]. No recovery of fertility has been observed in AR*w*P males tested up to 26 days.

Secondly, we know that the colonies of insects reared in the laboratory for subsequent field applications need to be periodically outbred to offset the effects of genetic adaptation to captivity and inbreeding depression [57-59]. For colonies of mosquitoes whose males are destined to be irradiated this problem is less relevant because there is no reproductive barrier between the insects of the colony and the wild types. Also for *Wolbachia* transinfected lines displaying a unidirectional CI pattern with uninfected wild populations [26-60] this problem does not arise because the males in nature are uninfected and therefore can be used to fertilize transinfected females.

In the case of AR*w*P or other transinfected mosquito lines displaying bidirectional CI towards wild populations (wild males are incompatible with females of the colony), the outbreeding procedures require the treatment of wild males with antibiotics for *Wolbachia* infection removal [32] and “compatibility” restore. The use of antibiotics, very useful at laboratory scale, may be rather laborious, time consuming and not cost-effective to produce large amounts of “antibiotic cured males” under mass rearing conditions. The discovery that AR*w*P females are partially fertile when mating with old wild *Ae. albopictus* males may simplify significantly the downstream efforts to preserve the genetic variability within the laboratory AR*w*P colonies; in fact, it would be sufficient to release periodically in the colony AR*w*P females mated with wild males aged at least 15 days.

In one hand the interest for the AR*w*P line in the IIT strategy against the Asian tiger mosquito has been increasing, in the other, new artificially generated infection types like *Ae. albopictus* harboring “*w*Mel” [29], showing anti-viral (Chikungunya and Dengue) properties associated to limited fitness costs, are promoting the application of “population replacement” strategies. Recent trends in the application of a population replacement program suggest that a phase of population suppression should be performed to support a following male-biased release of the avirulent invading mosquito strain (i.e. *w*Mel infected) minimizing any transient increase in disease risk or biting nuisance [61]. AR*w*P males, strong and persistent CI effectors, could be considered in the preliminary suppression phase of a population replacement program or for the application of suppression strategies in areas where there are not risks of pathogen transmission and consequently no need to replace a mosquito population. Although the AR*w*P mosquito line appears to be relatively robust and suitable for mass rearing, research is in progress to achieve a further attenuation of the negative effects of the new *Wolbachia* infection on female reproductive parameters (fecundity and fertility), as well as mating competitiveness of males, which is being evaluated not only in the laboratory (Moretti & Calvitti, unpublished data) but also in semi-natural (confined greenhouses) and field conditions.

## Competing interests

The authors declare that they have no competing interests.

## Author’s contribution

MC planned the work and performed Series I crossing experiments, analyzed data and wrote the first draft. RM contributed to the study design, performed crossing experiments and molecular analysis. ARS performed Series II of the crossing experiments and generated the HTA mosquito line. SLD contributed to the study design and supported the editing of the final manuscript. All authors approved the final version of the manuscript.
